# Molecular survey of tick-borne pathogens in domestic dogs from a rural region within the buffer zone of a conservation unit in the Brazilian Caatinga biome

**DOI:** 10.1590/S1984-29612025037

**Published:** 2025-06-30

**Authors:** Bruno Vinicios Silva de Araújo, Larissa Daniele Aires Oliveira do Carmo, Sthenia Santos Albano Amora, João Marcelo Azevedo de Paula Antunes, Francisco de Assis Leite Souza, Paulo Henrique Dantas Marinho, Juliana Fortes Vilarinho Braga

**Affiliations:** 1 Programa de Pós-graduação em Biociência Animal, Universidade Federal Rural de Pernambuco – UFRPE, Recife, PE, Brasil; 2 Médica Veterinária, Mossoró, RN, Brasil; 3 Programa de Pós-graduação em Ambiente, Tecnologia e Sociedade, Centro de Ciências Agrárias, Universidade Federal Rural do Semi-Árido – UFERSA, Mossoró, RN, Brasil; 4 Hospital Veterinário Universitário, Centro de Ciências Agrárias, Universidade Federal Rural do Semi-Árido – UFERSA, Mossoró, RN, Brasil; 5 Laboratório de Diagnóstico Animal, Departamento de Morfologia e Fisiologia Animal, Universidade Federal Rural de Pernambuco – UFRPE, Recife, PE, Brasil; 6 Escola Estadual de Educação Profissional Professora Maria Elsa Porto Costa Lima, Secretaria de Educação do Estado do Ceará –SEDUC, Aracati, CE, Brasil; 7 Universidade Federal do Piauí – UFPI, Campus Professora Cinobelina Elvas, Bom Jesus, PI, Brasil

**Keywords:** Anaplasma platys, Babesia vogeli, Ehrlichia canis, Hepatozoon canis, *Rhipicephalus sanguineus* sensu lato, environmental conservation units, Anaplasma platys, Babesia vogeli, Ehrlichia canis, Hepatozoon canis, *Rhipicephalus sanguineus* sensu lato, unidades de conservação ambiental

## Abstract

Although previous studies have identified *Ehrlichia canis*, *Anaplasma platys*, *Babesia vogeli*, and *Hepatozoon canis* in dogs in Northeastern Brazil, research on their presence within and around environmental conservation units remains scarce. The present study investigated the presence of tick-borne pathogens in domestic dogs in the rural region of Baraúna, within the buffer zone of the Furna Feia National Park (FFNP), an environmental conservation unit in the Caatinga biome of Rio Grande do Norte, northeastern Brazil. Blood samples from 52 dogs were collected to detect the presence of *A. platys* (16S rRNA), *B. vogeli* (18S rRNA), *E. canis* (16S rRNA), and *H. canis* (18S rRNA) DNA by Polymerase Chain Reaction (PCR). More than 90% of dogs were found to be infected with at least one pathogen, predominantly *E. canis* and *H. canis*. Co-infections (38.5%) and multi-infections with three (19.2%) and four (5.8%) pathogens were also frequent and diverse, underscoring the complexity of tick-borne diseases in this region. These findings highlight the epidemiological importance of dog-associated pathogens, and raise concerns regarding their potential transmission to wildlife within the conservation unit, as some of these pathogens have been previously described in wild mammalian species inhabiting the FFNP, including endangered species.

## Resumo

Apesar dos registros de *Ehrlichia canis*, *Anaplasma platys*, *Babesia vogeli* e *Hepatozoon canis* em cães no Nordeste do Brasil, estudos em unidades de conservação são limitados. Este estudo investigou a presença de patógenos transmitidos por carrapatos em cães domésticos na área rural de Baraúna, zona de amortecimento do Parque Nacional da Furna Feia (PNFF), unidade de conservação ambiental no bioma Caatinga do estado do Rio Grande do Norte, nordeste do Brasil. Amostras sanguíneas de 52 cães foram coletadas para pesquisa do DNA de *A. platys* (16S rRNA), *B. vogeli* (18S rRNA), *E. canis* (16S rRNA) e *H. canis* (18S rRNA) por meio da Reação em Cadeia da Polimerase. Mais de 90% dos cães estavam infectados por pelo menos um patógeno, predominantemente *E. canis* e *H. canis*. Coinfecções (38,5%) e multi-infecções por três (19,2%) e quatro (5,8%) patógenos foram frequentes e diversas, evidenciando a complexidade das doenças transmitidas por carrapatos na região. Esses achados destacam a importância epidemiológica de patógenos associados a cães e levantam preocupações sobre a possível transmissão para a vida silvestre na unidade de conservação, uma vez que alguns desses patógenos foram descritos anteriormente em espécies de mamíferos silvestres que ocorrem no PNFF, incluindo espécies ameaçadas de extinção.

## Introduction

Vector-borne emerging and re-emerging diseases represent a significant global public health challenge; however, despite their status as a major threat to public health worldwide they are commonly neglected, particularly in resource-constrained countries (Chala & Hamde, 2021; Mubemba et al., 2022), which can have significant implications for global health (Oliveira et al., 2020). Although more than 900 tick species are recognized worldwide, only approximately 10% of species are second to mosquitoes as major vectors of human and veterinary diseases (Tabor, 2024). Due to climate change and increased accessibility to certain environmental niches, the zoogeographical boundaries of ticks have been expanding in recent years (Han et al., 2016).

The various agents transmitted by ixodid ticks to which domestic dogs are susceptible include the bacteria *Ehrlichia canis* and *Anaplasma platys,* and the protozoa *Babesia vogeli* and *Hepatozoon canis*; indeed, hemoparasites are frequently encountered in these animals in Brazil (Dantas-Torres & Otranto, 2014; Malheiros et al., 2016; Sousa et al., 2017; Vieira et al., 2018). These hemoparasites are responsible for a variety of nonspecific clinical manifestations in infected hosts (Gonçalves et al., 2014; Bouzouraa et al., 2017), ranging from potentially fatal acute infections to recurrent chronic diseases (Kidd, 2019). Common clinical manifestations include fever, pale mucous membranes, anorexia, and weight loss, often accompanied by hematological abnormalities such as anemia, thrombocytopenia, and occasionally leukopenia (Cevidanes et al., 2023). These clinical signs tend to worsen when hosts are affected by multiple concurrent infections. Further, co-infection with more than one tick-borne pathogen is common, and has been associated with enhanced pathogenic processes and increased disease severity (Baneth, 2014).

Some of these pathogens are particularly important because of their potential for human infections, including *E. canis*, whose DNA has been detected in humans from Venezuela (Perez et al., 1996), Mexico (Silva et al., 2014), and Costa Rica (Bouza-Mora et al., 2017). Similarly, molecular evidence of *A. platys* in humans has been reported in both Venezuela (Arraga-Alvarado et al., 2014) and the USA (Maggi et al., 2013), suggesting the possible zoonotic potential of this agent.

Wildlife may act as a reservoir of arthropod-borne infections that can be transmitted to domestic animals and/or humans (Duscher et al., 2015). Among wild canids in Brazil, *E. canis* ribosomal RNA gene (*rrs*) has been detected in bush dogs (*Speothos venaticus*) and crab-eating foxes (*Cerdocyon thous*) (André et al., 2012; Sousa et al., 2017). Furthermore, within Brazil, an *Ehrlichia* spp. 16S rRNA genotype closely related to *E. canis* has been detected in various species of wild felids, including ocelots (*Leopardus pardalis*), jaguarundis (*Herpailurus yagouaroundi*), northern tiger cats (*Leopardus tigrinus*), pumas (*Puma concolor*), jaguars (*Panthera onca*), and lions (*Panthera leo*) maintained in captivity in zoos (André et al., 2010; André et al., 2012). In Brazil, *Ehrlichia* spp. 16S rRNA genotypes closely related to *Ehrlichia chaffeensis/E. canis* (Sousa et al., 2017) and a putative novel *Ehrlichia* sp. (‘*Candidatus* Ehrlichia dumleri’) (Perles et al., 2022) were detected in coatis (*Nasua nasua*) from midwestern Brazil. According to André (2018), both red foxes (*Vulpes vulpes*) and mustelids are exposed to *E. canis* in countries located in the Mediterranean Basin (Portugal, Spain, and Italy).

*Anaplasma* sp. 16S rRNA and *groEL* genotypes closely related to *Anaplasma bovis* were detected in *C. thous* in the Brazilian Pantanal and *S. venaticus* in a zoo in the state of São Paulo, respectively (Sousa et al., 2017; André et al., 2012). Additionally, *Anaplasma platys* has been detected in red foxes (*Vulpes vulpes*) in Portugal (Cardoso et al., 2015).

*Hepatozoon canis* is well adapted to its canine hosts (Baneth et al., 2003; Criado-Fornelio et al., 2006). Nonetheless, this may not be the case among wild canids from Brazil, where a *Hepatozoon americanum*-related genotype has been detected in crab-eating foxes (*Cerdocyon thous*) (Calchi et al., 2024).

A study conducted in the Pantanal wetland, midwestern Brazil, reported that 16.6% of domestic dogs tested positive for *Babesia vogeli* by PCR. Additionally, a high seropositivity rate (53.8%) to *B. vogeli* antigen was observed among free-living crab-eating foxes (*Cerdocyon thous*), although cross-reactions with other *Babesia* species could not be ruled out (Sousa et al., 2018). Indeed, a novel *Babesia* species (*Babesia pantanalensis*) has been detected in crab-eating foxes from the Brazilian Pantanal (Calchi et al., 2024).

Sang et al. (2021) reported the detection of *B. vogeli* for the first time in red foxes in Asia, and highlighted the potential for broader geographic distribution and the need for further investigation into the role of wild canids as hosts for these pathogens. In California (USA), it has been shown that wild coyotes (*Canis latrans*) can harbor both *Babesia vogeli* and *Babesia conradae*, which were also detected in coinfection, suggesting they may act as reservoirs for these pathogens, emphasizing the need to assess their role in the epidemiology of *B. vogeli* in domestic dogs (Javeed et al., 2022). Also, *Babesia* species are gaining growing attention as potential etiological agents of zoonotic diseases (Vannier et al., 2008; Young et al., 2019).

The Furna Feia National Park (FFNP) is an environmental conservation unit managed by the Chico Mendes Institute for Biodiversity Conservation (ICMBio), located between the municipalities of Mossoró and Baraúna in the Western region of Rio Grande do Norte state, Northeastern Brazil. With an area of ​​almost 8,500 ha, this park protects the rich biodiversity of the Caatinga and include a complex comprising more than 200 caves (ICMBio, 2017). The park harbors a significant portion of the medium- and large-mammal fauna of the Caatinga, including many threatened species and large frugivores that are locally extinct in much of the biome (Falcão et al., 2025).

The lack of understanding of the occurrence of tick-borne pathogens in domestic and wild animals in the region, along with the importance of preserving the area and conserving local biodiversity, highlights the need for studies to investigate the presence of pathogenic agents that may pose a risk to both domestic and wild animal populations. Furthermore, such research could provide a basis for developing more efficient wildlife protection and conservation strategies. In this context, the present study aimed to investigate the occurrence of tick-borne bacteria and protozoa in domestic dogs in the buffer zone of an important environmental conservation unit in the Brazilian Caatinga biome.

## Material and Methods

### Study area, dogs and samples

Blood samples from 52 dogs were analyzed. These samples were collected between March and April of 2022 in the community of Vila Nova I, located in the rural region of Baraúna (5°4′14″ S, 37°37′2″ W), Rio Grande do Norte state, Brazil. The community encompasses approximately 82,570 ha, and is situated at an altitude of 89 m, featuring a tropical climate with a dry season (Köppen-Geiger climate classification: As).

The study area, which is located in the buffer zone of the Furna Feia National Park, is less than 2.5 km from the limits of the strictly protected reserve. The Vila Nova I community is home to approximately 120 settled families and a population of over 100 dogs. Most of these dogs live freely and can roam alone or accompanied by their owners in the vicinity of the park, in addition to potentially having contact with wild animals in the surrounding area, since the settlement and the park are surrounded by remnants of native vegetation and plantations, environments also visited by the region's wildlife.

The animals included in the study were conveniently sampled during a polyvalent vaccination campaign targeting dogs in this community, without accounting for the presence of clinical signs, age, or breed. This vaccination campaign was organized by the Tiger Cat Conservation Initiative, and was part of the National Action Plan for the Conservation of Small Wild Cats (Brasil, 2022).

Of the 68 dogs vaccinated during the period, 24 were female and 44 were male, all at least 3 months old. The number of dogs sampled (52) is estimated to represent approximately 50% of the canine population in the community at the time of collection.

Samples were obtained by collecting 4 mL of blood via cephalic venipuncture, which was subsequently stored in tubes containing an anticoagulant (EDTA) and refrigerated until DNA extraction for subsequent PCR analysis. All procedures were approved by the Ethics Committee on Animal Use of the Universidade Federal Rural do Semi-Árido (UFERSA) under the protocol number 25/2022.

### DNA extraction and quantification

For DNA extraction from blood samples, the Genomic DNA mini Kit (Invitrogen®) was employed on 200 µL of whole blood sample, in accordance with the manufacturer's recommendations. Following extraction, DNA quality and concentration were assessed using a spectrophotometer (NanoDrop^TM^ Lite Thermo Scientific). Subsequently, DNA samples were stored at -20°C until PCR analysis.

### Pathogen DNA detection by Polymerase chain reaction

Polymerase chain reaction (PCR) targeting the dog *β-actin* gene was performed as an endogenous control. Specific primers and amplification programs validated in previously published studies were used to detect *A. platys* (16S rRNA), *B. vogeli* (18S rRNA), *E. canis* (16S rRNA), and *H. canis* (18S rRNA) (Table 1).

**Table 1:** Pathogen and target genes, oligonucleotide sequences, and sizes of PCR-amplified products of canine *β-actin*, *Anaplasma platys*, *Babesia vogeli*, *Ehrlichia canis*, and *Hepatozoon canis* were used in this study.

Pathogen and target gene	Primer	Oligonucleotide sequence	Product (bp)	Reference
		(5’-3’)		
Dog β-actin	Actb-F	GGCATCCTGACCCTGAAGTA	98	Turchetti (2014)
	Actb-R	CGCAGCTCGTTGTAGAAGGT		
				
*A. platys* (16S rRNA)	EPLAT5-F	TTTGTCGTAGCTTGCTATGAT	386	Matei et al. (2016)
	EPLAT3-R	CTTCTGTGGGTACCGTC		
				
*B. vogeli* (18S rRNA)	BAB1	GTGAACCTTATCACTTAAAGG	602	Duarte et al. (2008)
	BAB4	CAACTCCTCCACGCAATCG		
				
*E. canis* (16S rRNA)	EHO-F	AGAACGAACGCTGGCGGCAAGCC	478	Bulla et al. (2004)
	EHO-R	CGTATTACCGCGGCTGCTGGC		
	ECA-F	CAATTATTTATAGCCTCTGGCTATAGGAA	389	
	ECA-R	TATAGGTACCGTCATTATCTTCCCTAT		
				
*H. canis* (18S rRNA)	HC-18S-F	CACCAGGTCCAGACATAGAAAG	306	Kaur et al. (2020)
	HC-18S-R	AAGCTTACCAGCCAAGGTTAT		

PCRs were carried out in a final reaction volume of 25 μL, comprising 12.5 μL of master mix (Platinum PCR SuperMix, Cellco®), 1 μL of each primer (10 µM), 9.5 μL of DEPC water, and 1 µL of DNA. For the amplification of *E. canis* DNA, a nested PCR was employed, using 1 μL of the product from the first PCR step as a template for the second reaction. Amplification of *A. platys*, *B. vogeli*, and *H. canis* DNA was performed by conventional PCR. Known positive DNA samples for *Anaplasma platys* (JX437967), *Ehrlichia canis* (JX437966), *Babesia vogeli* (JX535812.1), and *Hepatozoon canis* were provided by partner laboratories were used as positive controls, while DEPC water was used as a negative control.

All PCR products were subjected to 1.5% agarose gel electrophoresis for 50 minutes at 120 volts in 1X Tris-borate-EDTA (TBE) buffer, using GelRed® as a DNA stain. To determine the size of the amplified products, a 100 bp molecular weight marker (Ludwig®) was used, following the manufacturer's recommendations. After electrophoresis, the gel was visualized using a UV light transilluminator (Proteinsimple®) and recorded using the AlphaImager Mini System software. Samples were considered positive for *A. platys*, *B. canis*, *E. canis*, or *H. canis* when amplified products of approximately 386, 602, 389, and 306 bp, respectively.

### Sequencing of PCR products

A total of one amplicon per pathogen was purified and sequenced. These PCR products corresponded to a single, clear band of the expected size in an agarose gel. These amplicons were purified using the PureLink™ Quick Gel Extraction and PCR Purification Combo Kit, following the manufacturer's instructions. Concentrations and purities were assessed via spectrophotometry (NanoDrop™ Spectrophotometers, Thermo Scientific, Waltham, USA). Sequencing was performed employing the Sanger method with species-specific primers in both directions. The sense and antisense sequences were trimmed and assembled using Geneious Prime software (version 2020.2.1). Subsequently, they were compared with sequences in the GenBank® nucleotide database using the BLAST® nucleotide tool to determine identity percentages. Partial 16S rRNA gene sequences (*A. platys* and *E. canis*) and 18S rRNA gene sequences (*B. vogeli* and *H. canis*) obtained in this study have been deposited in the GenBank database and accession numbers are shown in Results section.

### Data analysis

The data regarding the positivity of dogs for *A. platys*, *B. canis*, *E. canis*, and *H. canis* were tabulated in an Excel® spreadsheet for descriptive analysis using the absolute (n) and relative (%) frequencies.

## Results

**Figure 1:**
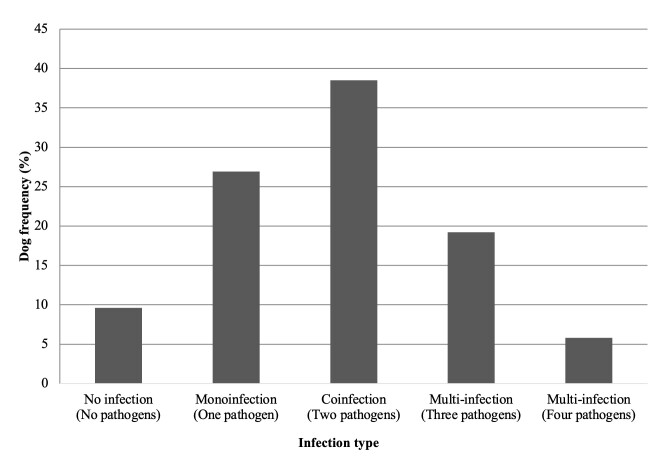
Frequency of no infection, mono-infection, co-infection, and multi-infection with *A. platys*, *B. vogeli*, *E. canis*, and *H. canis* in domestic dogs in the rural region of Baraúna, situated in Potiguar Caatinga, in 2022.

**Figure 2:**
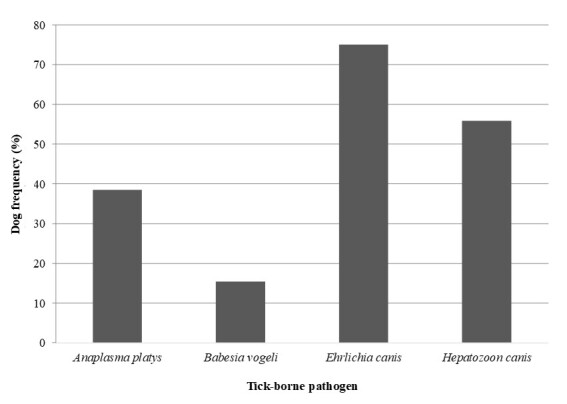
Frequency of PCR positivity for each investigated tick-borne pathogen in domestic dogs in the rural region of Baraúna, Potiguar Caatinga, in 2022.

A product of approximately 98 bp was amplified from all 52 samples by PCR to detect the canine *β-actin* gene, confirming the validity of the extracted DNA. Of the 52 dogs analyzed, 9.6% (9/52) were not infected with any of the pathogens (Figure 1), while DNA from at least one of the pathogens transmitted by *Rhipicehphalus sanguineus* sensu lato investigated in this study was detected in 90.4% (47/52) of the dogs. The DNA of *E. canis* and *H. canis* were detected most frequently, with positivity in 75% (39/52) and 55.8% (29/52) of the dogs analyzed, respectively (Figure 2). *Anaplasma platys* (38.5%, 20/52) and *B. vogeli* (15.4%, 8/52) were also detected, albeit in smaller numbers. Partial sequences of the *E. canis* 16S rRNA gene, *A. platys* 16S rRNA gene, *B. vogeli* 18S rRNA gene, and *H. canis* 18S rRNA gene obtained in this study have been deposited in the GenBank database under accession numbers PV354097, PV354061, PV354366, and PV354381, respectively. BLASTn analysis results of the sequences obtained from different pathogens are presented in Table 2.

**Table 2:** Pathogen species, GenBank accession numbers, and BLASTn results for DNA sequences obtained from canine blood samples collected in 2022 in the rural region of Baraúna, located in the Potiguar Caatinga.

**Scientific Name**	**Accession Number**		**Query Cover**	**E-value**	**% Identity**	**Sequence Size (bp)**
***Ehrlichia canis***						
***Ehrlichia sp.***	KY391799.1		100%	0.0	100.00%	387
***Ehrlichia canis***	OP268420.1		100%	0.0	100.00%	392
***Ehrlichia canis***	PP976541.1		100%	0.0	100.00%	413
***Ehrlichia canis***	OP268414.1		100%	0.0	100.00%	392
***Ehrlichia canis***	OP268418.1		100%	0.0	100.00%	392
***Ehrlichia canis***	OP268428.1		100%	0.0	100.00%	392
***Anaplasma platys***						
***Anaplasma sp.***	MT229115.1		100%	4e-180	100.00%	492
***Anaplasma platys***	MK736884.1		100%	4e-180	100.00%	427
***Anaplasma platys***	MN630835.1		100%	4e-180	100.00%	1446
***Anaplasma platys***	HE856819.1		100%	4e-180	100.00%	678
***Anaplasma platys***	PV545080.1		100%	4e-180	100.00%	421
***Anaplasma platys***	LC269820.1		100%	4e-180	100.00%	1487
***Babesia vogeli***						
***Babesia vogeli***	OM914863.1		100%	2e-163	100.00%	526
***Babesia vogeli***	KC616735.1		100%	2e-163	100.00%	602
***Babesia vogeli***	OM914868.1		100%	2e-163	100.00%	473
***Babesia vogeli***	PP716390.1		100%	2e-163	100.00%	591
***Babesia vogeli***	OM914862.1		100%	2e-163	100.00%	528
***Babesia vogeli***	MK881128.1		100%	2e-163	100.00%	811
***Hepatozoon canis***						
***Hepatozoon canis***	KC138531.2		100%	3e-156	100.00%	1693
***Hepatozoon canis***	MK091086.1		100%	3e-156	100.00%	1816
***Hepatozoon canis***	KC138532.2		100%	3e-156	100.00%	1687
***Hepatozoon canis***	PP494701.1		100%	3e-156	100.00%	997
***Hepatozoon canis***	MH615006.1		100%	3e-156	100.00%	7500
***Hepatozoon canis***	EU289222.1		100%	3e-156	100.00%	1529

**Figure 3:**
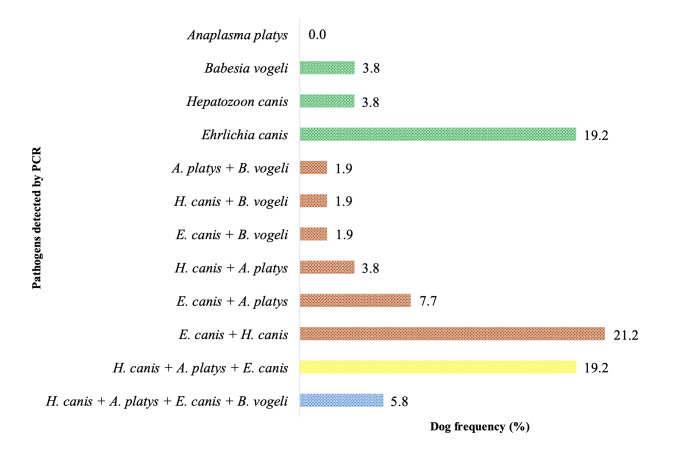
Frequency and profile of mono-infections^1^, co-infections^2^, and multi-infections with three^3^ or four^4^ tick-borne pathogens in domestic dogs in the rural area of Baraúna, situated in the Potiguar Caatinga, in 2022. ^1^Mono-infections: green bars ^2^Co-infection: orange bars ^3^Multi-infections with three pathogens: yellow bars ^4^Multi-infections with four pathogens: blue bar.

Regarding the type of infection, mono-infected dogs, represented by those in which only one of the pathogens had their DNA detected, accounted for 26.9% (14/52) (Figure 1). In this scenario, infection with *E. canis* was the most frequent (19.2%, 10/52) in dogs, followed by *H. canis* (3.8%, 2/52) and *B. vogeli* (3.8%, 2/52) (Figure 3). *Anaplasma platys* was not detected as a monoinfection in the analyzed animals.

In a representative subset of dogs (38.5%, 20/52), DNA from two agents was detected simultaneously, with the most frequent coinfections being those involving *E. canis*, as shown in Figure 3. Notably, the most frequently detected coinfections involved *E. canis* and *H. canis* (21.2%, 11/52), followed by *E. canis* and *A. platys* (7.7%, 4/52).

Multi-infection was also observed in 25.0% (13/52) of dogs. Three agents were detected simultaneously in 19.2% (10/52) of the animals, with all cases involving the same pathogens: *E. canis*, *H. canis*, and *A. platys* (Figure 3). Multiple infections with all four pathogens were diagnosed in three animals (5.8%, 3/52).

## Discussion

This study represents the first record of the occurrence of pathogens transmitted by *R. sanguineus* s.l. in domestic dogs in the rural area of Baraúna, located within the buffer zone of the protected area of FFNP. Our results demonstrated the circulation of *E. canis*, *H. canis*, *A. platys*, and *B. vogeli* in dogs throughout this region. Although other investigations have previously identified these pathogens in dogs in the Brazilian northeastern region (Rotondano et al., 2017; Lopes et al., 2018; Fonsêca et al., 2022; Nogueira et al., 2024), there is still a scarcity of research involving environmental conservation areas, such as the present study.

More than 90% of the animals were infected with at least one pathogen, while many (38.5%) were co-infected. The northeastern region of Brazil has favorable climatic conditions for the development of the tick vector *R. sanguineus* s.l. (Tanikawa et al., 2013), which increases the likelihood of exposure of these animals to a variety of pathogens transmitted by this vector (Rotondano et al., 2017; Lopes et al., 2018).

The results of the present study raise several concerns, as the blood samples were collected from apparently healthy dogs at the time of administration of the polyvalent vaccine. This suggests that many animals may be infected and untreated, a condition which favors pathogens’ transmission, highlighting the epidemiological importance of these dogs. During the subclinical phase of canine monocytic ehrlichiosis, the infected dog appears healthy but still acts as a carrier for *E. canis*. Furthermore, ticks can spread the bacteria even after 155 days following detachment from the host (Phillips, 2017).

This issue is particularly significant considering that the sampled dogs in this study reside in the buffer zone of a conservation unit that harbors significant and threatened mammalian fauna, while the potential for spillover of *E. canis* from domestic dogs to wildlife has already been suggested (André, 2018). The Caatinga, located in northeastern Brazil, is the largest dry tropical forest in the Americas (Banda-R et al., 2016), and is considered one of the 37 major wilderness areas remaining on the planet (Mittermeier et al., 2002). The Caatinga is home to 183 mammalian species, 11 of which are endemic, and 45 of which are classified as medium-to large-sized (Carmignotto & Astúa, 2017). Furna Feia National Park harbors a significant portion of the medium and large mammal fauna of the Caatinga, serving as a functional refuge for species regardless of the season (Falcão et al., 2025).

The bacterium *E. canis* was the most frequently detected pathogen in the domestic dogs analyzed (75.0%). Previous studies in Northeast region of Brazil have reported the prevalence of *E. canis* in domiciled dogs from different Brazilian cities. Also, in the state of Rio Grande do Norte, in the city of Mossoró, a study found *E. canis* to be the most prevalent pathogen (13.3%) in 120 dogs treated at a private veterinary clinic (Nogueira et al., 2024). In Ilhéus and Itabuna, Bahia, 7.8% of dogs presented at veterinary hospital were PCR positive (Carvalho et al., 2008), while in Recife, Pernambuco, 57% were PCR positive (Ramos et al., 2009). In Teresina, Piauí, the prevalence was 29.63% by PCR (Silva, 2010). Additionally, in Ilhéus, Bahia, 11% of dogs tested positive by PCR (Carlos et al., 2011).

In a prior study, Marinho et al. (2018) conducted a survey of medium and large mammals using camera traps in priority areas for biodiversity conservation in the Caatinga biome in Rio Grande do Norte, Brazil. The wild canine species observed in this area include crab-eating foxes (*Cerdocyon thous*), among which *E. canis* 16S rRNA has previously been detected in other Brazilian regions (André et al., 2012). Additionally, 16S rRNA genotypes closely related to *E. canis* have been reported in some wild felid species in the state of São Paulo, including ocelots (*Leopardus pardalis*), northern tiger cats (*Leopardus tigrinus*), jaguarundis (*Herpailurus yagouaroundi*), and pumas (*Puma concolor*) (André et al., 2010; André et al., 2012). It is worth noting that the latter three species are endangered and are targets of National Action Plans for the Conservation of Threatened Species (ICMBio, 2017).

Among the different members of the Procyonidae family, *Ehrlichia* spp. 16S rRNA genotypes closely related to *Ehrlichia chaffeensis/E. canis* (Sousa et al., 2017) have been previously detected in coatis (*Nasua nasua*) in the Brazilian Pantanal, state of Mato Grosso do Sul (Sousa et al., 2017). Members of this family, including the crab-eating raccoon (*Procyon cancrivorus*), are present in the Caatinga region of Rio Grande do Norte (Marinho et al., 2018), including FFNP (Falcão et al., 2025), highlighting the need for studies investigating the occurrence of this pathogen in these animals. This is particularly important considering that preventing the transmission of infections between domestic and wild carnivores is an important action of the National Action Plan for the Conservation of Threatened Species (Brasil, 2022).

It is also worth noting that Marinho et al. (2018) previously reported that, in addition to wild mammals, other species of mammals have been recorded in the evaluated priority areas for biodiversity conservation, including domestic dogs (*Canis lupus familiaris*), as well as inside the FFNP (Falcão et al., 2025), occasionally accompanied by humans. Given the close relationship between humans and their companion animals, particularly dogs, and the zoonotic nature of many tick-borne diseases, domestic carnivores may serve as sentinels for human infections, highlighting the importance of integrated surveillance and the role of veterinarians in addressing emerging public health threats (Beugnet & Marié, 2009).

*Hepatozoon canis* DNA was detected in 55.8% (29/52) of the dogs analyzed. In various regions of Brazil, studies have reported different prevalences of *H. canis* in domiciled and stray dogs. In Mossoró, Rio Grande do Norte, *H. canis* DNA was found in 6% of 120 dogs treated at a private veterinary clinic (Nogueira et al., 2024). In Recife, Pernambuco, 0.49% of domiciled dogs were PCR positive (Ramos et al., 2010). In Natal, Rio Grande do Norte, 10% of stray dogs tested positive by PCR (Lopes et al., 2018). Additionally, in the village of Jericoacoara, Ceará, 11.8% of domiciled dogs were PCR positive (Fonsêca et al., 2022). A *Hepatozoon* sp. 18S rRNA genotype closely related to *H. canis* was detected in an injured maned wolf (*Chrysocyon brachyrus*) in the state of São Paulo, southeastern Brazil (Perles et al., 2019).

In this study, *A. platys* (38.5%) and *B. vogeli* (15.4%) were detected, although at lower frequencies than the other agents investigated. In the Northeast region of Brazil, studies have revealed varying prevalence rates for both agents. In Mossoró, Rio Grande do Norte, Nogueira et al. (2024) found that 11.7% of domiciled dogs tested positive for *A. platys*, while in Teresina, Piauí, a study on domiciled dogs from a hospital population revealed that 41.48% tested positive by PCR (Silva, 2010). In Mossoró, Rio Grande do Norte, *B. vogeli* was found in 6% of dogs treated at a private veterinary clinic (Nogueira et al., 2024). In Jericoacoara, Ceará, 15% of domiciled dogs were PCR positive for *B. vogeli* (Fonsêca et al., 2022). In Chapadinha, Maranhão, 0.9% of domiciled dogs were PCR positive (Costa et al., 2015). In Patos, Paraíba, 10% of domiciled dogs were positive by PCR (Rotondano et al., 2015), while in São Luís, Maranhão, 7.69% were PCR positive (Galeno et al., 2018). In Teresina, Piauí, 4.8% of domiciled dogs tested positive by PCR (Braga et al., 2019).

Many of the dogs analyzed were found to be co-infected (38.5%) with multiple pathogens, mainly with *E. canis* and *H. canis*. In a recent study conducted by our research group on dogs from a hospital population in the city of Mossoró, municipality that contains part of the FFNP’s territory, we observed that 44.7% were mono-infected, while 21.5% presented coinfections and 6.1% showed multiple infections with these same agents (Araújo, 2024). In the present study, *E. canis* was the most frequently detected agent in animals (41.9%), followed by *H. canis* (35.4%), *A. platys* (21.5%), and *B. vogeli* (7.7%), which is similar to the findings in the rural areas of the present study.

All of these agents can cause a variety of clinical manifestations in infected hosts (Bouzouraa et al., 2017), with clinical signs ranging from potentially fatal acute infections to recurrent chronic diseases that can be transmitted by these canine hosts throughout their lives (Kidd, 2019). Specifically, *E. canis* is associated with a variety of clinical presentations, ranging from asymptomatic to severe disease (Sainz et al., 2015; Rodríguez-Alarcón et al., 2020). The second most frequently detected pathogen was *H. canis*, which contributed the most to the observed co-infection with *E. canis*. *Hepatozoon canis* typically presents with no clinical alterations in affected animals, or as relatively mild chronic infections (Baneth et al., 2003). However, this protozoan can cause severe clinical manifestations associated with high parasitic load (Baneth & Weigler, 1997) or concurrent infections (Harmelin et al., 1992). In endemic areas, different vector-borne pathogens can infect the same dog, thus exacerbating the clinical picture of the animal (Otranto et al., 2010).

One limitation of this study is the lack of additional data on the age, sex, breed, clinical signs, dogs' access to the street, contact with other animals and the number of animals with ectoparasites, as well as the lack of tick collection at the time of blood sampling, despite many animals being visibly infested. Additionally, it was not feasible to test for infections caused by other pathogens, such as canine distemper virus, parvovirus, or *Leishmania* spp. These factors highlight the complexity of correlating ectoparasite presence with infection status and the challenges in addressing co-infections, emphasizing the need for comprehensive diagnostic approaches in future studies.

It is worth noting that the blood samples used in this study were collected during vaccination campaigns. This is important as vaccines should be administered only to healthy animals, as immunosuppressive changes have been observed in the immune systems of healthy dogs following polyvalent vaccination (Strasser et al., 2003). The high percentage of infected animals at the time of vaccination raises questions regarding the adequacy of the response to antigenic stimulation, as well as the development of protective antibody titers. This compromise in vaccine response could contribute to the increased susceptibility of these dogs to infection following the eventual exposure to pathogens included in polyvalent vaccination, or even greater susceptibility to disease development by the current tick-borne infection due to potential post-vaccinal immunosuppression (Strasser et al., 2003). As such, it is recommended to perform diagnostic tests for tick-borne pathogens to provide specific treatment before vaccination of dogs from the region. Further studies are required to contribute to the seroepidemiological knowledge of these agents in dogs.

## Conclusions

This study is the first to document tick-borne pathogens in domestic dogs in the rural area of Baraúna, located within the buffer zone of a conservation unit in the Brazilian Caatinga. The results showed a high prevalence of *E. canis*, *H. canis*, *A. platys*, and *B. vogeli*, with over 90% of dogs infected with at least one pathogen. These findings highlight the importance of dogs as potential hosts for the transmission of tick-borne pathogens, necessitating further research and targeted health measures to protect both animal and wildlife populations in the region.

## Data Availability

Data will be made available on request.
